# Perceptions of nursing informatics on quality of nursing practice and competency among nurses in Jordan

**DOI:** 10.3389/fdgth.2025.1585603

**Published:** 2025-07-02

**Authors:** Rami Alnajjar, Amr Zyoud, Hanan Al-Shamaly, Abdelrhman Tamimi, Moath AbedRabbu

**Affiliations:** ^1^ Clinical Nursing Department, Applied Science Private University, Amman, Jordan; ^2^ Primary Department, Al-Ahliyya Amman University, Amman, Jordan; ^3^ Nursing Department, Middle East University, Amman, Jordan; ^4^ Nursing Department, King Saud University Medical City, Riyadh, Saudi Arabia

**Keywords:** nursing informatics, electronic health records, clinical nursing practice, nurses' competency, EMR (electronic medical record)

## Abstract

**Background:**

The application of nursing informatics and its specific components, such as the EHR/EMR comes with an array of positive implications. However, these benefits are poorly explored in Jordan as a country where digitized nursing information still struggles through challenges.

**Objectives:**

This research was conducted to examine the perceptions on the nursing informatics towards quality of clinical nursing practice, and to determine how nursing informatics affect clinical competency among nurses.

**Methods:**

Quantitative research was conducted between September 2018 and May 2023. Data was collected using a research questionnaire administered to 256 clinical nurses in three government hospitals in Amman government, Jordan. The hospitals that were part of the study are the following: Prince Hussein bin Abdullah Hospital, Al-Bashir Hospital and Dr. Jamil Al-Totangi Hospital. Analysis of data was done using SPSS (version 26) and SPSS-AMOS.

**Results:**

The respondents expressed that nursing informatics ensures a quality of clinical nursing practice in clinical nursing care plan, use of nursing informatics, quality of patient care, clinical nurse's competency and patient safety. Further statistical analysis showed that that there is a statistically significant effect of nursing informatics on the competency of clinical nurses (*p* = .001; *β* = .547).

**Conclusion:**

Nursing informatics has various significant benefits that relate to quality of clinical practice and nurses' competency, as perceived by the Jordanian clinical nurses.

**Recommendation:**

Comprehensive adoption of nursing informatics in Jordanian hospitals may help improve the overall quality of patients' care.

## Introduction

Universal health coverage (UHC) is one of the foundation of the Sustainable Development Goals (SDGs), specifically highlighted in Target 3.8 of SDG 3. Achieving UHC necessitates a fundamental transformation in how healthcare is defined and delivered, with a strong focus on improving access, quality, and efficiency (1). In this context, digital health technologies have emerged as powerful tools to support this shift, offering innovative solutions to address longstanding challenges in healthcare systems.

Indeed, digital health technologies continue to advance and evolve, they play a growing role in shaping a more efficient, responsive, and patient-centered healthcare system (2). Furthermore, nurses are the backbone and the largest labor force that play an integral part in healthcare services that directly influences the quality of healthcare besides the well-being of patients, families, and communities (3).

Nursing plays a pivotal role in employing information systems and digital health technologies serving as essential contributors to evidence-based practice through tools such as Electronic Health Records (EHRs) to ensure providing support to achieve effective practice and meet patient needs through the field of Nursing Informatics, to ensure that technological solutions reflect nursing perspectives and enhance the quality of care (4, 37).

Nursing informatics, a is a sub element of medical informatics, which encompasses nursing expertise with digital information and communication technologies to support the management of health information (5). Its goal is to enhance the quality of nursing services that influence the well-being of clints via enhancing how nursing data is utilized in care delivery.

Nursing informatics in East and Southeast Asia, as well as the Middle East has played a crucial role in advancing digital health solutions and supporting frontline care particularly throughout the COVID-19 pandemic by facilitated the shift toward distance nursing education, supported e-learning for continued professional development, and enabled the use telemedicine services and the use of digital tools to inform and educate local communities (38). These initiatives demonstrate how nursing informatics has become an essential component in strengthening healthcare response and resilience during public health emergencies.

Having undergone a continuous improvement and evolution for decades, the application of nursing informatics took a significant stance in the early 1990s when various healthcare organizations shifted from analogous paper-based information processing to the electronic fashion (6). The applications continued to gain more attention from the healthcare practitioners and even organizations like the “Institute of Medicine” (IOM), which have encouraged the electronic processing of healthcare information (7).

Within the domain of nursing informatics, various concepts and functionalities have emerged with overlapping roles. For instance, electronic health records (EHRs) or electronic medical records (EMR) emerged as a robust instrument for saving and handling patients' medical data (8). HER refers to the use of automated devices to document and store clinical information of a patient or their treatment journey (6, 9). However, the scope and depth of adoption and application of the electronic health records may vary with region or country.

According to the bibliometric analysis conducted by Luan et al. (10), there has been a steady increase in the application and citations of the EHR. Indeed, more than 2600 publications were noted overall, between 2000 and 2020 (10). Nevertheless, studies conducted in the developing countries like Jordan have also cited critical challenges and barries that affect the adoption of EHR, such as financial capacity, inadequate information system and alignment with the current system without causing interruptions (11).

Furthermore, the application of nursing informatics, such as the EHR/EMR comes with an array of positive implications already highlighted in the literature. For instance, the promoting nurses' cognitive capacity (12), economic incentives (13), patients' risk exposure analysis (34), practical research and decision making (14), and nursing diagnosis (15). Consequently, this has reflected in critical significance in improving the competency of information processing among the healthcare team (8).

Nursing informatics serves as a foundational element for effective EHR and plays a vital role in enhancing patient care. Notably, nursing informatics has been recognized as a key factor in strengthening clinical performance and improving the overall quality of healthcare services (16). However, Evidence from the literature indicates that nurses face some challenges with the regular usage of the HER in Jordan and other developing countries (17, 18). Despite its importance, limited research has focused on nursing informatics and its inclusive influence on the excellence of care delivered that is mold by the perceptions of nurses in Jordan (11, 18).

In Jordan, The Hakeem program is the national electronic health record system, which is design to enhance healthcare quality, and linking public health services (16). Established in 2012 by the Ministry of Health in Jordan, it now operates in 196 facilities and covers 77% of public hospital beds, supporting various healthcare functions and is used by over 30,000 professionals, storing more than seven million patient records (17). Therefore, this research was conducted to examine the perceptions on the nursing informatics towards quality of clinical nursing practice, and to determine by what means nursing informatics affect nursing clinical competences.

## Methods

The quantitative methodology was applied in the study; wherein statistical data was collected for analysis using the relevant statistical tools. The study was conducted between September 2018 and May 2023, and the data was collected between January 2021 and March 2021 from three government hospitals in Amman government, Jordan. The hospitals that were part of the study are the following: Prince Hussein bin Abdullah Hospital, Al-Bashir Hospital and Dr. Jamil Al-Totangi Hospital. These hospitals were conveniently selected since they have adopted active nursing informatics in their clinical operations via Hakeem program (17, 19).

The sample size was determined using the online calculator, Raosoft Software, which ended up with a total of 256 participants. These participants were to be selected from the three hospitals proportionately depending on their population sizes. Accordingly, 49 participants were selected from Prince Hussein Hospital, 151 from Al-Bashir Hospital and 56 from Al Totangi Hospital.

Data was collected by using a survey questionnaire at the respective hospitals. The questionnaire was newly designed and was hence piloted for validity and reliability. There were two sections. The first section gathered the quantitative aspects of the participants' variables such as age, gender, and level of education. The second section had questions that seek to establish how participants regard nursing informatics as well as five variables of quality of nursing practice.

Analysis of data was done using SPSS version 26 and SPSS-AMOS. Accordingly, three statistical tools were applied: the descriptive statistics, and multiple linear regression SPSS version 26, and Structural Equation Model of SPSS- AMOS were also used. Interpretations of the analysis were based on each objective.

## Results and discussion

A total of 256 participants were involved in this study. From this number, 111 (43.4%) were males and 145 (56.6%) were females. One hundred and forty-three (59.9%) were under 40 years old, and 113 (44.1%) were over 40 years old. Greater part of the study participants specifically, 126 (49.2%) had an undergraduate (bachelor) degree while 81 (31.6%) had diploma and 49 (19.1%) had postgraduate degree. Concerning the units of operation, there was a relatively fair distribution of the participants in outlined hospital units; 41 (16%) in the intensive care, 31 (12.1%) in emergency care, 42 (16.4%) in the medical—surgical unit, 21 (8.2%) in the operation rooms and the highest number, 51 (19.9%) were in the ob-gyn Unit. A total of 11 participants (4.3%) were in the cardiac intensive care unit, 19 (7.4%) worked in paediatric unit, and 20 (7.8%) worked in the outpatient clinic (Table 1).

**Table 1:** Participants sociodemographic features.

Variable	*N* (%)
Age	
<40	143 (55.9)
>40	113 (44.1)
Gender	
Male	111 (43.4)
Female	145 (56.6)
Education level	
College diploma	81 (31.6)
University undergraduate	126 (49.2)
Postgraduate degree	49 (19.1)
Unit of operation	
Intensive care unit	41 (16)
Emergency care unit	31 (12.1)
Medical—Surgical unit	42 (16.4)
Operation rooms unit	21 (8.2)
Obe-gyn unit	51 (19.9)
Cardiac intensive care unit	11 (4.3)
Paediatric unit.	19 (7.4)
Out Patient clinics	20 (7.8)
Others	20 (7.8)

To determine nurses' perceptions on the nursing informatics towards quality of clinical nursing practice, the variable was measured by the summation of 5 ordinal variables to compute nursing informatics, which provided the participants “various views on the implications of nursing informatics on the various individual concepts that describe quality of nursing practice”.

Regarding the use of informatics to augment healthcare service delivery, 24.2% of the respondents strongly agreed that nursing informatics help to improve healthcare service delivery (Table 2). Under the section of enhancement of information quality, the largest proportion (39.5%) strongly agreed that the use of nursing informatics help to improve quality of clinical information (Table 2). Further, 36.7 per cent strongly agreed that nursing informatics enhances satisfaction among clinical (Table 2). Overall, higher proportions either agreed or strongly agreed to the items that nursing informatics help to improve nursing care activities.

**Table 2:** Perception on nursing informatics.

Quality of clinical nursing practice	Strongly disagree (%)	Disagree (%)	Neutral (%)	Agree (%)	Strongly agree (%)
Augments quality of healthcare service delivery	19.9	12.5	10.9	32.4	24.2
Enhances information quality	12.5	11.5	4.3	32	39.5
Enhances satisfaction the among clinical	19.9	11.7	4.3	27.3	36.7
Enhances healthcare service quality	28.1	3.9	3.5	40.2	24.4
Promotes teamwork among clinical nurses	16.4	19.9	8.2	23.8	31.6

The observation that nursing informatics helps improve nursing care activities is also apparent in literature, since a number of previous studies have equally reported the same observation (20, 21). The benefits of nursing informatics in specific nursing care activities, such as teamwork, information quality and quality of healthcare services have also been noted before (22).

The research also presented specific questionnaire items about the impact of nursing informatics on the quality of clinical nursing practice. In this case, the quality of clinical nursing practice was described by nursing care plan, usability features, quality of patient care, nurses' competencies and patients' safety.

Regarding the significance of nursing care plan, this study found out that a greater percentage of the study participants (36.7%) strongly agreed that nursing informatics improves the assessment of the patients' medical result/obtain information from patients “medical history to help create a plan. Also, 28.1 per cent of the respondents strongly agreed that nursing informatics provide diagnoses benchmarks for an effective care plan by the clinical nurses”. Almost half of the participants (49.2%) strongly agreed that nursing informatics provides baseline information of the patients ‘care for evaluation and subsequent follow up. Then, 24.2 per cent of the respondents strongly agreed that nursing informatics provides a retrievable plan for recording patients' expected outcome, and lastly, 25 per cent strongly agreed that nursing informatics facilitate easier recording of the nursing care initiatives for future implementation (Table 3).

**Table 3:** Nursing care plan.

Role of nursing informatics in nursing care plan	Strongly disagreed (%)	Disagree (%)	Neutral (%)	Agree (%)	Strongly agree (%)
Enhances assessment of the patients' medical result/obtain information from patients' medical history to help create a plan	19.9	8.2	12.5	22.7	36.7
Provide diagnoses benchmarks for an effective care plan by the clinical nurses.	14.8	11.7	8.2	37.1	28.1
Facilitates easier recording of the nursing care initiatives for future implementation.	23	24.2	20.7	7	25
Provides a retrievable plan for recording patients expected outcome.	10.9	10.9	7.8	46.1	24.2
Provide baseline information of the patients' care for evaluation and subsequent follow up	15.6	15.2	8.6	11.3	49.2

The application of nursing informatics in nursing care activities was also noted for specific usability features, including data entry (37.9% strongly agreed), alerting clinical activities (28.1% strongly agreed), and availing clinically relevant information (28.5% strongly agreed) (Table 4).

**Table 4:** Usability of nursing informatics.

Usability of nursing informatics	Strongly disagree (%)	Disagree (%)	Neutral (%)	Agree (%)	Strongly agree (%)
Data entry	15.2	3.5	11.7	31.6	37.9
Alerting	28.1	12.1	8.2	36.7	14.8
Visual display	19.5	12.5	16.4	27.7	23.8
Availability of information	23.8	7.8	12.9	27	28.5
System automation and default	19.1	7.8	12.9	27	28.5

Regarding the usability features of nursing informatics elements, such as the EHR, Booth et al. (22) express that nursing practice will have to rely on the digital practice as a way of keeping up with the evolutionary changes that comes with technological improvements. The introduction of artificial intelligence would also have a smooth incorporation into the digital activities in nursing practice. Digitized patients' information is also enhanced by the appealing visual display of the systems, and the automated functions with reminders that help improve efficiency in the delivery of nursing services (18). Nevertheless, other scholars have also acknowledged that nursing informatics helps in easier documentation practices for better nursing service delivery (23).

Quality of patient care is another significant feature examined under the role of nursing informatics. A larger proportion of the participants (32%) strongly disagreed that nursing informatics improves timely diagnosis (7.8%) and facilitates patients' assessments for therapeutic adjustments (33.3%) (Table 5). Studies show that nursing informatics, through the EMR helps to avail patients' information, such as their history for quick judgments and decisions about diagnosis (24). Moreover, it was also noted that almost a half of the participants (48%) agreed that nursing informatics helps in ensuring patients safety and 4.3% percent strongly agreed that nursing informatics help to make patients' assessments for appropriate medical adjustments (Table 5).

**Table 5:** Role of nursing informatics in improving quality of patient care.

Quality of patient care	Strongly disagree (%)	Disagree (%)	Neutral (%)	Agree (%)	Strongly agree (%)
Timely diagnosis.	32	24.6	7.8	27.7	7.8
Convenient medical care.	27.3	8.2	12.5	16.8	35.2
Patient safety.	3.1	11.3	8.6	48	28.9
Assessment for adjustments	33.2	20.7	12.1	29.7	4.3
Speed of access to medical care	18.4	12.1	11.7	21.1	36.7

Regarding nurses' competency as a concept within quality of nursing care practice, this study established that 44.1 percent of the participants strongly agreed that nursing informatics improves emotional skills among the clinical nurses. The relationships between use of digitized system and nurses' emotional wellbeing can be related to the easy that comes with the use of systems like EMR (25). According to Weinschreider et al. (26) the applications of EHR in nursing care helps to improve nurses' attitudes, which can also be linked to the emotional ease that comes along their applications. Kinnunen et al. (27) directly associated the ease of use in the EHR as a significant feature that enhances the competency among the clinical nurses.

Additionally, this study observed that another comparatively large percentage of the participants (41%) strongly agreed that nursing informatics enhances the ability of clinical nurses to understand their patients' needs. This benefit comes from the ability to quicky access patients' information in order to identify their medical and clinical needs (24). Accordingly, the clinical nurses can adjust their patients' medication and treatment plans in a way that factors in their medication history, including allergies and their preferred medications.

Other notable significance includes facilitating clinical decisions, where 39.1 per cent of the participants strongly agreed, and empowering nurses where 45.3 per cent of the participants agreed (Table 6). The EHRs help clinical nurses to make better decisions as they provide a comprehensive view of patients' scenario that covers medical history, medications, allergies, test results, and imaging all in one place (28, 29). Moreover, empirical evidence shows that EHRs can help to improve nurses' confidence and empowerment by improving workflow, communication and patient education (30, 31).

**Table 6:** Role of nursing informatics in enhancing nurse’s competency.

Nurses Competency	Strongly disagree (%)	Disagree (%)	Neutral (%)	Agree (%)	Strongly agree (%)
Abilities to understand needs	14.8	10.9	3.9	29.3	41
Improve emotional skills	16.4	4.3	3.1	32	44.1
Collaborative work	10.9	10.0	11.7	29.3	37.1
Clinical decisions	16.4	4.3	3.1	37.1	39.1
Empowers nurse	7	3.9	7.4	45.3	36.3
Professionalism	11.3	10.9	7.4	50	20.3

In terms of patient safety, notable observations were made in the provision of faster access to accurate patients' information where 61% strongly agreed, and reduction of medication errors, where 39% of the participants strongly agreed to the idea (Table 7). However, when it comes to the reduction of incidences of transfusion reaction count, majority of the participants (51%) remained neutral (Table 7). In the previous studies, studies have noted that medication errors help to reduce chances of medication errors and other unwanted drug administration mistakes (29, 32). In another previous investigation, Jindal and Raziuddin (33), p. 84) noted that EHR helps to “reduces medical errors, wrong site surgery, improper dosage delivery to a patient, wrong medication, etc. by 50–60 per cent”. Accordingly, nursing informatics is significant in reducing the medication mistakes and enhancing patients' wellbeing, which connotates patients' safety.

**Table 7:** Role of nursing informatics in enhancing patients' safety.

Patients' safety	Strongly disagree (%)	Disagree (%)	Neutral (%)	Agree (%)	Strongly agree (%)
Reduces incidences of medication errors	3	13	11	34	39
Enhances timely assessment of patient safety predictors	7	24	46	17	5
Reduces incidences of transfusion reaction count	1	5	51	32	11
Provides faster access to accurate patients' information	2	11	7	19	61
Reduces incidences of postoperative sepsis	13	24	24	7	32

Finally, this study sough to determine how nursing informatics affect the clinical competency among nurses. To establish the relationship, a simple linear regression was conducted, which showed that there is a statistically significant effect of nursing informatics on the competency of clinical nurses (*p* = .001; *β* = .547) (Table 8).

**Table 8:** Coefficient of association between nursing informatics and clinical nurses' competency (95% CI).

Variable	Beta	t	Sig.	F	R square
Constant		10.906	.001	108.578	
Nursing informatics	.547	10.420	.001		.299

From the statistical outcomes in Table 8, this study noted that nursing informatics has a statistically significant relationship with nurses' competency. The positive relationship can be linked to various concepts. First, nursing informatics, through EHR enables efficient access to extensive patients' medical databases and research, ensuring nurses stay current with evidence-based practices (8). Also, nursing informatics skills facilitate data interpretation from various sources such as patient monitors and lab results, aiding in trend identification, early disease detection and risk assessment (9, 34).

Overall nursing informatics also equips nurses with proficiency in electronic health records, enabling accurate documentation, access to historical patient data, and utilization of clinical decision support tools (13). Nurses proficient in nursing informatics can integrate emerging technologies like telehealth and mobile apps to enhance communication, medication adherence, and remote monitoring, thereby improving patient care and outcomes (35). Lastly, Wisner et al. (12) observed that nursing informatics fosters critical thinking and problem-solving skills among nurses, empowering them to analyse data critically and contribute effectively to care plans even in the emerging diseases (14, 36).

However, it must be noted that this study incurred a limitation that needs considerations by the readers and before policy implementation. The notable limitation was on data collection from the specific hospitals that had a well-established electronic health record system. There are possibilities of false positive outcomes since these three hospitals have been utilizing the Hakeem system for a long time, and the level of utilization might not give a generalizable picture of the state of electronic health records in all hospitals in Jordan.

## Data Availability

The raw data supporting the conclusions of this article will be made available by the authors, without undue reservation.
